# The Role of the Atypical Kinases ABC1K7 and ABC1K8 in Abscisic Acid Responses

**DOI:** 10.3389/fpls.2016.00366

**Published:** 2016-03-24

**Authors:** Anna Manara, Giovanni DalCorso, Antonella Furini

**Affiliations:** Department of Biotechnology, University of VeronaVerona, Italy

**Keywords:** ABA, ABC1K proteins, abiotic stress, germination, senescence, stomatal closure

## Abstract

The ABC1K family of atypical kinases (activity of *bc1* complex kinase) is represented in bacteria, archaea, and eukaryotes. In plants they regulate diverse physiological processes in the chloroplasts and mitochondria, but their precise functions are poorly defined. ABC1K7 and ABC1K8 are probably involved in oxidative stress responses, isoprenyl lipid synthesis and distribution of iron within chloroplasts. Because reactive oxygen species take part in abscisic acid (ABA)-mediated processes, we investigated the functions of *ABC1K7* and *ABC1K8* during germination, stomatal movement, and leaf senescence. Both genes were upregulated by ABA treatment and some ABA-responsive physiological processes were affected in *abc1k7* and *abc1k8* mutants. Germination was more severely affected by ABA, osmotic stress and salt stress in the single and double mutants; the stomatal aperture was smaller in the mutants under standard growth conditions and was not further reduced by exogenous ABA application; ABA-induced senescence symptoms were more severe in the leaves of the single and double mutants compared to wild type leaves. Taken together, our results suggest that ABC1K7 and ABC1K8 might be involved in the cross-talk between ABA and ROS signaling.

## Introduction

The ABC1K family of atypical kinases is characterized by a highly conserved ABC1 domain and one or more protein kinase domains (Leonard et al., 1998). This ancient family is highly conserved among bacteria, archaea and eukaryotes, and has undergone substantial expansion in photosynthetic organisms (Lundquist et al., 2013). The strong conservation of ABC1K proteins suggests they play a fundamental biological role. The prototypical member is ABC1/COQ8, a nuclear-encoded protein identified in the yeast *Saccharomyces cerevisiae* which is required for ubiquinone biosynthesis in the mitochondria. In yeast, this protein is needed for aerobic respiration at the mitochondrial *bc1* complex level, and its inactivation makes the *bc1* complex unstable resulting in a respiratory defect (Bousquet et al., 1991). The biological role of ABC1/COQ8 homologs has been investigated in other species revealing a conserved functional role in ubiquinone synthesis in bacteria, archaea and the mitochondria of eukaryotes (Ernster and Forsmark-Andrée, 1993; Macinga et al., 1998; Poon et al., 2000; Do et al., 2001; Iiizumi et al., 2002; Mollet et al., 2008). However, little is known about the role of ABC1K homologs in chloroplasts.

The *Arabidopsis thaliana* genome contains 17 members of the ABC1K family. The first to be characterized, the mitochondrial ABC1At protein, differs only slightly from *S. cerevisiae* ABC1/COQ8 and partially restores the respiratory defect in *abc1^-^* mutant yeast (Cardazzo et al., 1998). AtOSA1 was the first chloroplast-localized ABC1K protein to be investigated; it is induced by cadmium (Cd) and oxidative stress, and it does not complement the yeast *abc1^-^* mutant, suggesting a functional difference between this protein and mitochondrial ABC1 (Jasinski et al., 2008). Another ABC1K protein, AtACDO1, was also shown to be localized in chloroplasts, and is associated with chlorophyll degradation and oxidative stress responses under high-light (Yang et al., 2012a). Two plastoglobule-localized ABC1K proteins, ABC1K1 and ABC1K3, regulate prenylquinone metabolism, which plays an important role in plant stress responses and chloroplast morphology (Lundquist et al., 2013; Martinis et al., 2013). ABC1K1 is needed to stabilize chlorophyll-binding proteins in photosynthetic complexes, and *abc1k1* knockdown plants show defects in sugar metabolism suggesting that ABC1K1 may integrate photosynthesis with associated metabolic pathways in chloroplasts (Martinis et al., 2014). Recently, it was reported that another chloroplast localized ABC1 gene, AtSIA1 together with AtOSA1, with which it shows high sequence conservation, participates in iron distribution inside the chloroplast and in plant response to oxidative stress (Manara et al., 2014).

The *A. thaliana* plastoglobule proteome was shown to include six of the eight ABC1K proteins currently known to be localized in chloroplasts (Ytterberg et al., 2006; Vidi et al., 2007; Lundquist et al., 2012b, 2013). Similarly, the proteomic analysis of maize (*Zea mays*) leaves indicated that eight ABC1K proteins are located in the chloroplasts (Majeran et al., 2010). Indeed, *in silico* predictions of protein localization indicate that most of the maize, rice (*Oryza sativa*) and *A. thaliana* ABC1K proteins are located in either the chloroplasts or the mitochondria, and this may potentially be the case in all plants (Lundquist et al., 2012a). A systematic nomenclature based on phylogeny has been proposed for the ABC1K family to avoid the assignment of arbitrary names, and we have adopted this nomenclature herein (Lundquist et al., 2012a).

In a previous study, we characterized ABC1K7 (formerly AtSIA1) and ABC1K8 (formerly AtOSA1) to determine their physiological functions (Manara et al., 2014). Among the *A. thaliana* ABC1K proteins, ABC1K7 was most closely related to ABC1K8 (46% identity) and showed 50% identity with cyanobacterial ABC1K proteins such as *Crocosphaera watsonii* ZP00517317 suggesting this protein was also a member of the chloroplast ABC1K group. The strong sequence conservation between ABC1K7 and ABC1K8 prompted us to study the phenotypes of *abc1k7* and *abc1k8* single mutants and *abc1k7/abc1k8* double mutant (to investigate potential functional redundancy) as well as transgenic lines overexpressing ABC1K7 and ABC1K8 in their mutant backgrounds (Manara et al., 2014). This confirmed the chloroplast localization of ABC1K7 as previously reported for ABC1K8 (Jasinski et al., 2008) and revealed its role in oxidative stress responses, isoprenyl lipid synthesis and (together with ABC1K8) iron distribution within the chloroplast (Manara et al., 2014). Plant ABC1K proteins have previously been associated with different forms of abiotic stress tolerance (Jasinski et al., 2008; Gao et al., 2010, 2012; Wang et al., 2011). The phenotypes of the *abc1k7* and *abc1k8* single mutants and *abc1k7/abc1k8* double mutants supported this hypothesis because they were less tolerant to ROS and the antioxidant network was activated even under standard growth conditions (Manara et al., 2014). In addition, an untargeted lipidomic analysis demonstrated that ABC1K7 and ABC1K8 are required for chloroplast lipid synthesis or accumulation and modulate chloroplast membrane lipid composition (Manara et al., 2015). *abc1k7* and *abc1k8* single mutants produced lower levels of the highly unsaturated lipid digalactosyldiacylglycerol (DGDG) than WT plants and also different forms of monogalactosyldiacylglycerol (MGDG) and kaempferol. The *abc1k8* mutant is also characterized by higher levels of oxylipin-conjugated DGDG and sinapates, and the *abc1k7/abc1k8* double mutant produced even higher levels of oxylipin-conjugated MGDG and DGDG (Manara et al., 2015).

Reactive oxygen species can modulate ABA-induced seed dormancy (Liu et al., 2010) and stomatal closure (Neill et al., 2008; Simontacchi et al., 2013). The accumulation of ROS may also cause the decline in ABA levels during germination, e.g., H_2_O_2_ accumulates rapidly during seed imbibition and can induce ABA catabolism and the biosynthesis of gibberellins (Liu et al., 2010). ROS, especially H_2_O_2_, act as secondary messengers during ABA-induced stomatal closure (Zhang et al., 2001). ABA is a hormonal trigger of leaf senescence (Gepstein and Thimann, 1980) and ABA levels in leaves increase during this process (Zhao et al., 2010), which can be promoted by the application of exogenous ABA (Weaver et al., 1998).

To gain insight into the role of ABC1K7 and ABC1K8 in the regulation of ABA signaling during seed dormancy, stomatal responses and leaf senescence, we studied the hormonal regulation of ABC1K7 and ABC1K8 and their ability to regulate other genes. We found that the *ABC1K7* and *ABC1K8* genes are upregulated by ABA treatment after 24 h and that the proteins play a significant role in ABA-mediated physiological processes. ABC1K7 and ABC1K8 regulate several ABA-responsive genes that control germination, abiotic stress responses, stomatal closure and leaf senescence. There were no significant differences in endogenous ABA levels among *abc1k7* and *abc1k8* mutants, *abc1k7/abc1k8* double mutants and WT plants, but the mutants were more sensitive to exogenous ABA treatments. Our results suggest there is substantial crosstalk between ROS-mediated and ABA-mediated signaling pathways in plants.

## Materials and Methods

### Plant Material and Growth Conditions

The *abc1k7* (*atsia1*) mutant (SALK_020431), the generation of the *abc1k7/abc1k8* (*atsia1/atosa1*) double mutant and the generation of transgenic lines *OX-ABC1K7* and *OX-ABC1K8* overexpressing ABC1K7 and ABC1K8, respectively, were reported in an earlier publication (Manara et al., 2014). The *A. thaliana abc1k8* (*atosa1*) mutant seeds (GABI_132G06) were kindly provided by Prof. E. Martinoia (City University of Zurich, Switzerland; Jasinski et al., 2008). The *OX-ABC1K7* and *OX-ABC1K8* transgenic lines were used in the complementation experiments to confirm differences between mutant and WT plants. Three independent lines for both *OX-ABC1K7* and *OX-ABC1K8* were chosen and the expression of the recombinant fusion proteins was confirmed by western blot (Manara et al., 2014). These overexpressing lines were obtained in mutant backgrounds and they show complementation of mutant phenotype. As described in Manara et al. (2014) the different overexpressing lines complemented the mutant phenotype in a similar manner and, for simplicity only one overexpressing line for each gene was showed in this work. *A. thaliana* ecotype Col-0 was used as the WT control.

Plants were grown under controlled conditions in a phytochamber (16 h light/8 h dark, illumination 100–120 μmol m^-2^s^-1^, day/night temperature 22°C/18°C). Seeds were sown in Petri dishes on water-soaked Whatman filter paper and incubated for 3 days at 4°C in the dark. The plants were then transferred to soil in a phytochamber under the controlled conditions described above.

### Abiotic Stress, ABA, and ABA Synthesis Inhibitor Treatments

To analyze the effect of other abiotic stresses on gene expression, seedlings grown *in vitro* on MS medium (Murashige and Skoog, 1962) with 0.7% agar and 3% sucrose were transferred to the following different conditions for 0, 1, 2, 5, and 24 h and kept under the growth conditions described: (i) ½-strength Hoagland solution (Hoagland and Arnon, 1938) supplemented with 10 μM CdSO_4_; (ii) plates directly kept at 4°C; (iii) ½-strength Hoagland solution supplemented with 300 mM mannitol. Leaf samples were collected after 5 and 24 h treatment, and immediately frozen in liquid nitrogen. The effect of ABA treatment on the *ABC1K7* and *ABC1K8* expression levels was analyzed in *A. thaliana* WT seedlings grown in ½-strength Hoagland solution supplemented with ABA (20 μM). As in the previous experiments, leaf samples were collected after 5 and 24 h treatment, and immediately frozen in liquid nitrogen. The analysis of *ABC1K7* and *ABC1K8* expression upon treatment with the ABA synthesis inhibitor NDGA was conducted in *A. thaliana in vitro* grown WT plantlets, which were transferred into Petri dishes containing disks of Whatman paper soaked with liquid Murashige and Skoog (MS) medium solution (control condition) and liquid MS containing 100 μM nordihydroguaiaretic acid (NDGA, Sigma-Aldrich; Ren et al., 2007). Treated plantlets were incubated under controlled conditions in a phytochamber (16 h light/8 h dark, illumination 100–120 μmol m^-2^s^-1^, day/night temperature 22°C/18°C). Leaf samples were collected after 2, 5, and 24 h treatment, and immediately frozen in liquid nitrogen.

### Germination Analysis

Sterilized seeds were germinated on MS agar medium supplemented with different concentrations of ABA (0, 20, 50, and 100 μM, for 2 and 4 days), mannitol (100 and 200 mM, for 2 days), and NaCl (100 mM for 3 days and 200 mM for 7 days). The plants were grown in the phytochamber under the controlled conditions described above. Approximately, 100 seeds of each genotype were sown on each plate and scored for germination. Each experiment was performed in triplicate.

### RNA Extraction, cDNA Synthesis, and Real-Time RT-PCR

Total RNA was extracted from fresh or liquid nitrogen frozen tissues using TRIzol reagent (Invitrogen, Karlsruhe, Germany). After DNase treatment, first-strand cDNA was synthesized using SuperScript^TM^ III Reverse Transcriptase (Invitrogen). Real-time RT-PCR (40 amplification cycles) was carried out using the ABI PRISM^®^ 7000 Sequence Detection System (Applied Biosystems, Foster City, CA, USA) with Platinum^®^ SYBR^®^ Green qPCRSuperMix UDG (Invitrogen). Each reaction was carried out in triplicate and melting curves were analyzed to ensure the amplification of a single product. Quantitative data were normalized to the mean of two endogenous reference genes: actin2/8 (*At3g18780/At1g49240*; primers 1 and 2) and ubiquitin 5 (*At3g62250*; primers 3 and 4). The 2^-ΔΔCT^ method for the analysis of relative gene expression levels was used to organize the data (Livak and Schmittgen, 2001). *ABC1K7* (*At3g07700*) and *ABC1K8* (*At5g64940*) transcription levels were quantified using specific primers (primers 5 and 6 for *ABC1K7*, and primers 7 and 8 for *ABC1K8*). *SAG12*mRNA levels were also measured (primers 9 and 10). The levels of ABA-regulated gene transcripts *KIN2* (*COR6.6*; *At5g15970*; primers 11 and 12), *HAB1* (*At1g72770*; primers 13 and 14), *ABI1* (*At4g26080*; primers 15 and 16), *COR15b* (*At2g42530*; primers 17 and 18), *KIN1* (*At5g15960*; primers 19 and 20), and *ERD10* (*At1g20450*; primers 21 and 22) were also measured. All primers are listed in **Table 1** and their efficiency was determined using LinRegPCR v2012.2 (Ramakers et al., 2003).

**Table 1:** Primer sequences.

Gene	ATG number	Primer number	Nucleotide sequence (5′–3′)	Primer efficiency
β*-actin 2/8*	*At3g18780/ At1g49240*	1	AACATTGTGCTCAGTGGTGG	1,957
		2	GACCTTAATCTTCATGCTGCT	
*Ubiquitin5*	*At3g62250*	3	AGCATAAGAAGGTTAAGCTCG	1,993
		4	TCCACAGGTTGCGTTAGGG	
*ABC1K7*	*At3g07700*	5	GAACTCGATTCAGGCGATC	1,914
		6	TCCTCCAAGAACTGTATACAT	
*ABC1K8*	*At5g64940*	7	TAGAGTCTGAGAGGGCATTT	1,985
		8	AGAATTCAGATACAAAATTGTT	
*SAG12*	*At5g45890*	9	GAAAGTGGATATATGAGGATTC	1,897
		10	AGCTTAACACGGTTTTGAATTC	
*KIN2* (*COR6.6*)	*At5g15970*	11	CGCAACAGGCGGGAAAGAG	1,904
		12	ACTCCCAAAGTTGACTCGGAT	
*HAB1*	*At1g72770*	13	GTCCATCGGTGACAGATATCT	1,876
		14	AAGACCGTCACTGGCTAGTAT	
*ABI1*	*At4g26080*	15	TGGCGGTTCTCAGGTAGCG	1,853
		16	TCCAGCCACGTATCACCATC	
*COR15b*	*At2g42530*	17	CAACGAAGCCACAAAGAAAGC	1,943
		18	GCTTCAATGGTTTTCTCAACAAT	
*KIN1*	*At5g15960*	19	GCTGGCAAAGCTGAGGAGAA	1,823
		20	CCGCATCCGATACACTCTT	
*ERD10*	*At1g20450*	21	TTCTTCCTCTTCGAGTGATGAA	1,876
		22	TCTCTTCTTCCACTGTTTTCAC	


### Measurement of Stomatal Apertures

Detached rosette leaves from illuminated plants (100–120 μmol m^-2^s^-1^) grown in hydroponic solution with or without the addition of 20 μM ABA were fixed overnight (0.1% glutaraldehyde, 4% paraformaldehyde, 0.1 M sodium phosphate buffer pH 7.2) and the stomata were observed directly using a Leica DM RB microscope (Leica Microsystem GmbH) as previously described (Mustilli et al., 2002). At least 50 stomata were measured for each genotype and condition. The stomatal aperture was measured using ImageJ software (http://rsbweb.nih.gov/ij/).

### Analysis of ABA-Induced Leaf Senescence

Detached rosette leaves were incubated in water or in ABA solution (20 and 50 μM) for 5 days under the conditions described above, as previously reported (Jia et al., 2013). Each experiment was performed in triplicate.

### Determination of Chlorophyll Content

Leaves were weighed, frozen and ground to powder under liquid nitrogen. Chlorophyll was extracted in 80% acetone saturated with Na_2_CO_3_, and cell debris was removed by centrifugation (10,000 × *g*, 4°C, 10 min). Absorbance values were recorded at 750.0, 646.6, and 663.3 nm. The concentrations of chlorophyll *a* and *b* were determined using classical equations (Porra, 2002). Each experiment was performed in triplicate.

### Measurements of ABA Content

Abscisic acid content was measured in leaves, dry seeds and imbibed seeds of plants grown in control conditions and treated for 7 days with 100 mM mannitol and 100 mM CdSO_4_. Plant material has harvested and immediately frozen in liquid nitrogen. Grinded tissues have been processed, and ABA quantification has been obtained, accordingly to the protocol previously described in Glauser et al. (2014).

### Statistical Analysis

All experiments were carried out at least three times. Differences between WT, mutants and overexpressing lines were determined by one-way analysis of variance (ANOVA). Significant differences are indicated as follows: ^∗^*P*<0.05, ^∗∗^*P*<0.01, ^∗∗∗^*P*<0.001.

## Results

### Analysis of *ABC1K7* and *ABC1K8* Expression in Response to Abiotic Stresses, Exogenous ABA, and upon Inhibition of Endogenous ABA Synthesis

**FIGURE 1:**
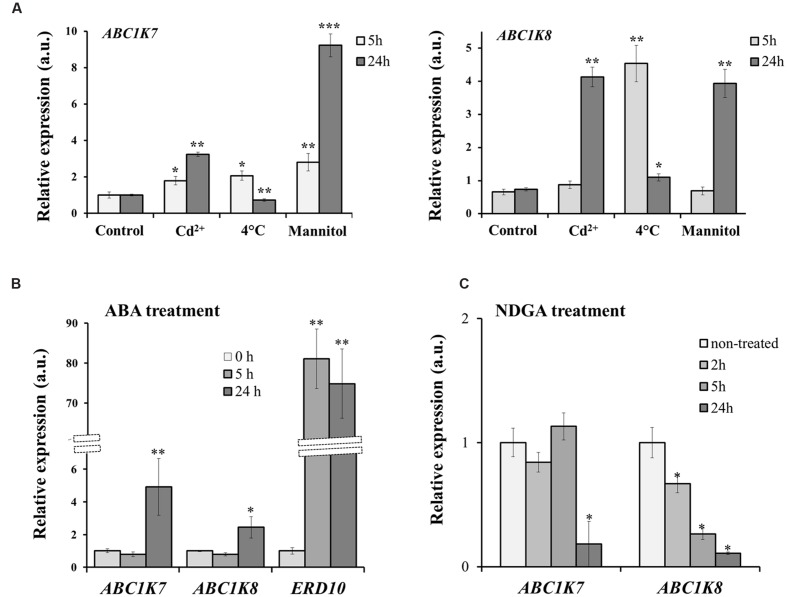
**Analysis of *ABC1K7* and *ABC1K8* mRNA levels.** **(A)** Real-time RT-PCR analysis of *ABC1K7* and *ABC1K8* in the leaves of WT plants exposed to a variety of abiotic stress. **(B)** Real-time RT-PCR analysis of *ABC1K7*, *ABC1K8*, and *ERD10* in the leaves of WT plants exposed to 20 μM ABA for different durations. **(C)** Real-Time RT-PCR analysis of RNA abundance of *ABC1K7* and *ABC1K8* genes upon treatment with the ABA synthesis inhibitor NDGA for 2, 5, and 24 h. The expression levels were calculated using the 2^-ΔΔCT^ method and are relative to the control leaf expression level (0 h). In **C**, the expression levels are relative to the expression in control plantlets, which was set to 1 for each gene. Each value represents the mean ± SE. Significant differences relative to the control treatment are shown as follows: ^∗^*P* < 0.05 and ^∗∗^*P* < 0.01, ^∗∗∗^*P* < 0.001.

We investigated the effect of different forms of abiotic stress on the expression of *ABC1K7* and *ABC1K8* by submitting plants to Cd treatment, cold and mannitol treatment. As reported in **Figure 1A**, although differently, both genes are upregulated in response to the stress: *ABC1K7* and *ABC1K8* show a substantial and clear upregulation upon Cd and mannitol treatments lasting also 24 h after exposure, while cold stress imposes only an early response (5 h upon treatment). Because ABA acts as an endogenous messenger in plant stress responses (Hirayama and Shinozaki, 2007) and induces changes in gene expression (Rabbani et al., 2003; Shinozaki et al., 2003), we also measured the levels of *ABC1K7* and *ABC1K8* mRNA in the leaves of WT plants with and without exposure to exogenous ABA. As control of ABA effect on gene expression, we included in the analysis *ERD10*, an ABA responsive gene. As highlighted in **Figure 1B**, both genes were induced by exposure to ABA for 24 h, with significance levels of *P* < 0.01 for *ABC1K7* and *P* < 0.05 for *ABC1K8*. The *ABC1Ks* ABA-mediated induction was also reported in rice and poplar (Gao Q.S. et al., 2011; Wang et al., 2013). Interestingly, the application for 24 h of 100 μM NDGA, an inhibitor of the endogenous ABA biosynthesis (Ren et al., 2007; Gao G. et al., 2011), induced a downregulation in expression of both *ABC1K7* and *ABC1K8* (**Figure 1C**). Notably, the expression of *ABC1K8* is responding to inhibition of ABA synthesis already after 2 h of treatment (**Figure 1C**).

### The Role of ABC1K7 and ABC1K8 during Germination

**FIGURE 2:**
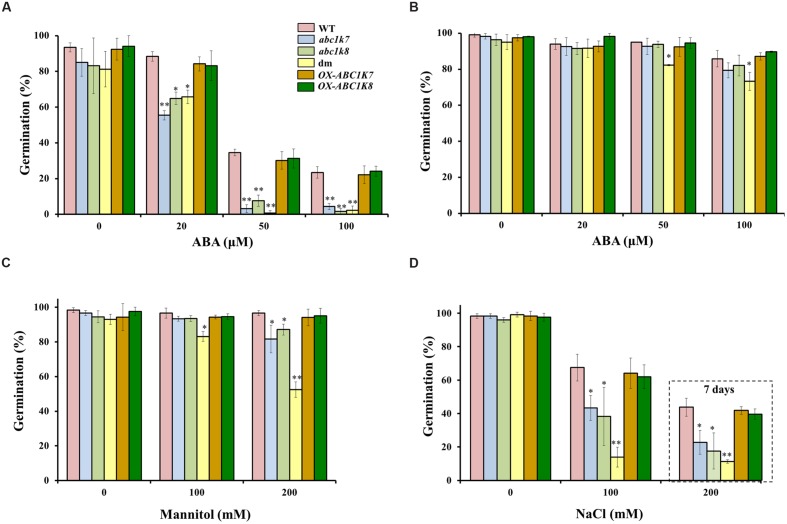
**Analysis of ABA, mannitol and NaCl treatment on the germination of WT, mutant, and overexpressing lines.** **(A,B)** Effect of ABA (0, 20, 50, and 100 μM) exposure for **(A)** 2 days and **(B)** 4 days after sowing. **(C)** Effect of mannitol (0, 100, and 200 mM) 2 days after sowing. **(D)** Effect of NaCl (0, 100 mM) 3 days after sowing and (200 mM) 7 days after sowing (dashed box). Each value represents the mean ± SD. Approximately, 100 seeds from each genotype were analyzed in three independent experiments. Significant differences relative to the WT are shown as follows: ^∗^*P* < 0.05 and ^∗∗^*P* < 0.01.

We investigated the role of *ABC1K7* and *ABC1K8* in the ABA-mediated regulation of germination by comparing the effect of increasing ABA concentrations (Umezawa et al., 2009) on the germination of *abc1k7* and *abc1k8* single mutants, *abc1k7/abc1k8* double mutants, overexpressing lines and WT plants. After 2 days of exposure to 20 μM ABA, the percentage of germinating plants was significantly lower in the *abc1k7* (*P* < 0.01), *abc1k8* (*P* < 0.05), and *abc1k7/abc1k8* (*P* < 0.05) lines compared to WT plants, and this effect was more significant in all three mutant lines (*P* < 0.01) when the ABA concentration was increased to 50 and 100 μM (**Figure 2A**). After 4 days of exposure to ABA, the mutant plants showed partial recovery and the only significant difference in germination frequency (*P* < 0.05) was found between the *abc1k7/abc1k8* double mutant and WT plants at the highest ABA concentration tested (**Figure 2B**). OX-ABC1K7 and OX-ABC1K8 overexpressing lines show a WT-like behavior confirming the complementation of the phenotype (**Figure 2**). There were no differences between WT and mutant plants in terms of leaf or root growth on media supplemented with either 50 or 100 μM ABA (data not shown). However, these conditions resulted in general growth inhibition and leaf yellowing in all genotypes (data not shown). Next we tested the effect of osmotic stress and salt stress on the germination of mutant and WT plants by exposing the seeds to mannitol or sodium chloride. Osmotic stress induced by 100 or 200 mM mannitol did not affect the germination of WT plants but the lower of the two concentrations significantly (*P* < 0.05) reduced the frequency of germination in the double mutant. The higher concentration significantly (*P* < 0.05) reduced the frequency of germination in both the *abc1k7* and *abc1k8* single mutants, and had a more severe effect (*P* < 0.01) on the double mutant (**Figure 2C**). Moderate salt stress (100 mM NaCl) affected the frequency of germination in all genotypes 3 days after sowing although there was a significantly greater effect in the *abc1k7* and *abc1k8* mutants (*P* < 0.05) which became more severe in the *abc1k7/abc1k8* double mutant (*P* < 0.01). Higher salt stress intensity (200 mM NaCl) affected the frequency of germination in all genotypes 7 days after sowing although again there was a significantly greater effect in the *abc1k7* and *abc1k8* mutants (*P* < 0.05) which became more severe (*P* < 0.01) in the *abc1k7/abc1k8* double mutant (**Figure 2D**).

### The Role of ABC1K7 and ABC1K8 during Stomatal Closure

**FIGURE 3:**
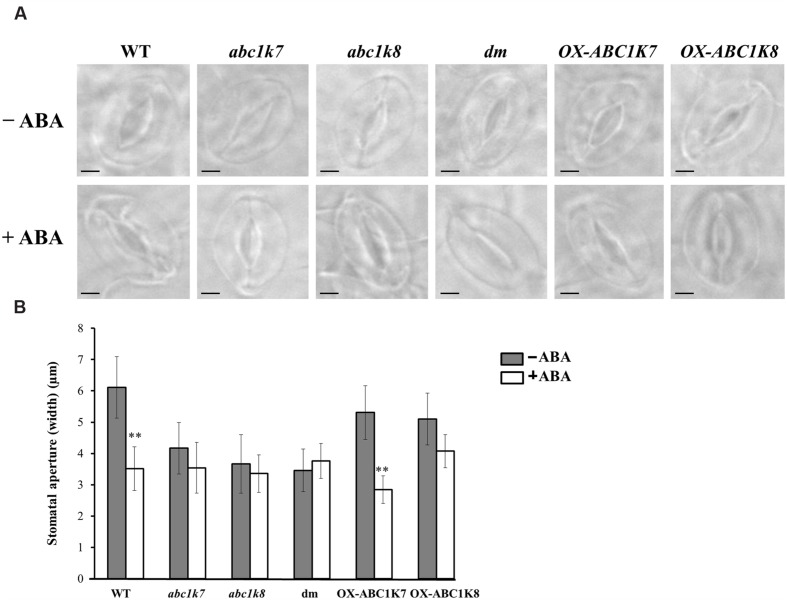
**Effect of ABA treatment on the stomatal aperture.** **(A)** Microscope images of stomata from WT, mutant and overexpressing plants under standard growth conditions (– ABA) or following 5 h exposure to 20 μM ABA (+ ABA). Bars = 5 μm. **(B)** Measurement of the stomatal apertures of WT, mutant and overexpressing plants under standard growth conditions (– ABA) or following 5 h exposure to 20 μM ABA (+ ABA). Each value represents the mean ± SD. Approximately 50 stomata from each genotype were analyzed in three independent experiments. Significant differences relative to the WT are shown as ^∗∗^*P* < 0.01.

The role of ABC1K7 and ABC1K8 in the ABA-mediated stomatal response was tested in *abc1k7* and *abc1k8* mutants, *abc1k7/abc1k8* double mutants, transgenic lines overexpressing each of the proteins (*OX-ABC1K7* and *OX-ABC1K8*) and WT controls (Manara et al., 2014). Following exposure to light (100–120 μmol m^-2^s^-1^) in the absence of ABA, the stomatal aperture of the mutant and double mutant lines was significantly smaller (*P* < 0.01) than that of WT plants and overexpressing lines (**Figures 3A,B**). ABA treatment (20 μM for 5 h) induced stomatal closure in the WT and overexpressing lines, whereas the stomata aperture in *abc1k7* and *abc1k8* mutants and *abc1k7/abc1k8* double mutants was not further reduced by ABA application (**Figures 3A,B**). The stomatal density and size under normal conditions were similar in the WT, *abc1k7*, *abc1k8*, and *abc1k7/abc1k8* plants (data not shown).

### The Role of ABC1K7 and ABC1K8 in ABA-Induced Leaf Senescence

**FIGURE 4:**
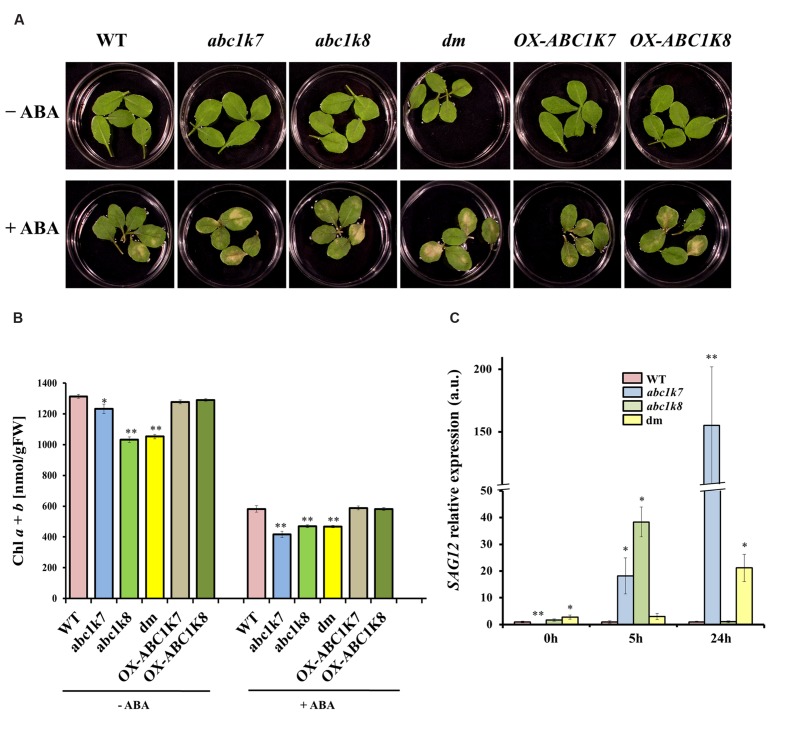
**Analysis of ABA-induced leaf senescence.** **(A)** Leaves detached from WT, mutant and overexpressing plants were floated on sterile water (– ABA) or 50 μM ABA for 5 days (+ ABA). **(B)** Chlorophyll content of detached leaves treated with sterile water (– ABA) or 50 μM ABA for 5 days (+ ABA). Each value represents the mean ± SD. **(C)** Real-time RT-PCR analysis of *SAG12* in the leaves of WT and mutant plants under standard growth conditions (– ABA) or following 5 or 24 h exposure to 20 μM ABA (+ ABA). WT *SAG12* expression was set to 1 in both control and ABA treatment. Each value represents the mean ± SE. Significant differences relative to the WT are shown as follows: ^∗^*P* < 0.05 and ^∗∗^*P* < 0.01.

The effect of ABA-induced leaf senescence was investigated using leaves detached from the mutant, double mutant and overexpressing plants as well as WT controls. Leaves were floated on water or 50 μM ABA for 5 days and senescence was assessed by observing leaf yellowing and measuring the chlorophyll content (**Figure 4**). Detached leaves floated on water showed no visible yellowing regardless of the genotype, whereas those floated on ABA solution developed yellow patches after 5 days. The leaves from *abc1k7*, *abc1k8*, and *abc1k7/abc1k8* mutant plants development more extensive senescence symptoms than leaves from the WT and overexpressing plants, the latter behaving similar to WT (**Figure 4A**).

These qualitative observations were measured objectively by determining the chlorophyll content of the leaves before and after ABA treatment. The total chlorophyll concentration was significantly lower (*P* < 0.01 and *P* < 0.05) in the mutant leaves compared to the leaves of WT and overexpressing plants, even when the leaves were floated on water (**Figure 4B**). This agrees with the previously observed phenotypes of *abc1k8* and *abc1k7*/*abc1k8* mutants, which were paler green and contained less chlorophyll than WT plants under controlled conditions (16 h light/8 h dark, 100–120 μmol m^-2^s^-1^; Manara et al., 2014). When the detached leaves were floated on 50 μM ABA, there was loss of chlorophyll in all genotypes, but the loss was highly significant (*P* < 0.01) in the *abc1k7* and *abc1k8* mutant and *abc1k7/abc1k8* double mutant lines (**Figure 4B**). The impact of the *abc1k7*, *abc1k8*, and *abc1k7/abc1k8* mutations on leaf senescence was evaluated further by measuring the abundance of the senescence-associated transcript *SAG12* (**Figure 4C**). Under control condition *SAG12* transcription is almost null in *abc1k7* mutant respect to WT (*P* < 0.01) but significantly higher levels in the *abc1k7/abc1k8* double mutant (*P* < 0.05). After exposure to ABA for 5 h, there was a significant increase in *SAG12* mRNA levels in both the *abc1k7* and *abc1k8* mutant lines compared to WT (*P* < 0.05) and a highly significant increase (*P* < 0.01) in the *abc1k7* mutant after exposure to ABA for 24 h (**Figure 4C**).

### Analysis of ABA-Regulated Gene Expression

The role of ABC1K7 and ABC1K8 in ABA signaling was investigated by measuring the abundance of several ABA-modulated transcripts in *abc1k7*, *abc1k8*, and *abc1k7/abc1k8* mutant lines compared to WT controls. The target genes comprised two type-2C phosphatases, *ABI1* (Leung et al., 1994) and *HAB1* (Rodriguez et al., 1998), which are negative regulators of ABA signaling; two non-seed LEA genes, *Cor15b* (*LEA23*; Wilhelm and Thomashow, 1993) and *ERD10* (*LEA5*; Kiyosue et al., 1994); and two genes that are induced by cold, ABA, dehydration and mannitol, *KIN1* (Wang et al., 1995) and *KIN2* (Kurkela and Borg-Franck, 1992).

**FIGURE 5:**
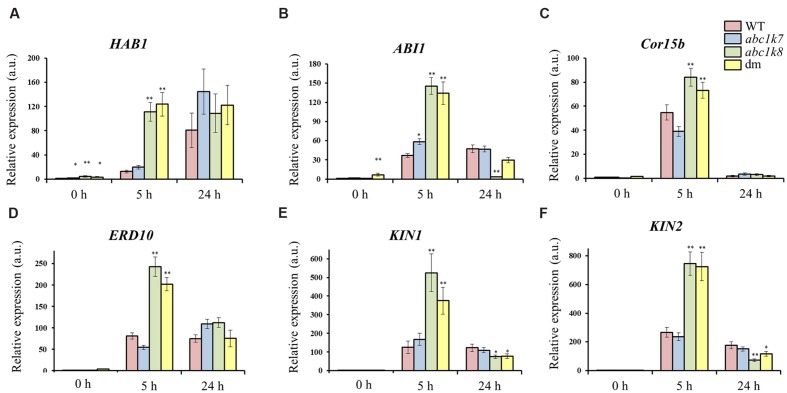
**Analysis of ABA-regulated gene expression.** Real-time RT-PCR analysis of **(A)** *HAB1*, **(B)** *ABI1*, **(C)** *Cor15b*, **(D)** *ERD10*, **(E)** *KIN1*, and **(F)** *KIN2* expression in the leaves of WT and mutant plants before (0 h) and after (5 and 24 h) exposure to 20 μM ABA. Each value represents the mean ± SE. Significant differences relative to the WT are shown as follows: ^∗^*P* < 0.05 and ^∗∗^*P* < 0.01.

All six genes were induced by ABA in all genotypes (**Figure 5**). However, following 5 h of ABA exposure, *HAB1* was more strongly induced (*P* < 0.01) in the *abc1k8* and *abc1k7/abc1k8* lines (**Figure 5A**) and *ABI1* was more strongly induced all the mutant lines (*P* < 0.01 and *P* < 0.05) compared to WT plants (**Figure 5B**). These differences disappeared when ABA exposure was prolonged to 24 h (**Figures 5A,B**). Similarly, both LEA genes were significantly induced (*P* < 0.01) in the *abc1k8* and *abc1k7/abc1k8* lines following exposure for 5 h but the differences disappeared after 24 h (**Figures 5C,D**). Finally, *KIN1* and *KIN2* expression was significantly induced (*P* < 0.01) by ABA treatment for 5 h in the *abc1k8* and *in abc1k7/abc1k8* lines but significantly suppressed (*P* < 0.01 and *P* < 0.05) by exposure for 24 h (**Figures 5E,F**). No significant differences between mutant and WT plants were observed in the expression of *LEA24* and *LEA38*, two other LEA genes with chloroplast-localized products (data not shown).

### Analysis of Expression of ABA Metabolism Correlated Genes

To explore the link between the ABA-mediate response and the increased stress sensitivity showed by *abc1k7* and *abc1k8*, the expression of genes involved in ABA metabolism was analyzed by Real Time PCR, comparing the expression level in control conditions and after a stress of 6 h dehydration (see Supplementary Material). Two genes involved in ABA synthesis, *AAO3* (the Abscisic Aldehyde Oxidase 3) and *NCED3* (9-*cis*-Epoxycarotenoid-Dioxygenase 3; Kanno et al., 2012) and two genes coding for proteins that were shown to have a role in ABA transport, *AIT1* (ABA-Importing Transporter 1) and *DTX50* (Detoxification Efflux Carrier 50; Zhang et al., 2014) were analyzed. As shown in **Supplementary Figure S1**, with the exception of *AIT1*, which is not modulated by dehydration, the other genes analyzed are overexpressed in WT by this stress condition. This stress, imposes an upregulation of *DTX50*, *AAO3*, and *NCED3* also in *abc1k7*, *abc1k8* mutant and *abc1k7/abc1k8* double mutant plants. Notably the transporter protein *DTX50* is upregulated in mutant lines already in control conditions (**Supplementary Figure S1**). Furthermore, *NCED3*, coding a key enzyme of ABA biosynthesis, is constitutively expressed in mutant backgrounds. *AAO3* transcription level in *abc1k7* and *abc1k8* is similar to that of WT, whereas in the *abc1k7/abc1k8* double mutant, the mRNA level upon standard growth conditions drops down, but it is normally up-regulated by dehydration.

### Analysis of the Endogenous ABA Content

**FIGURE 6:**
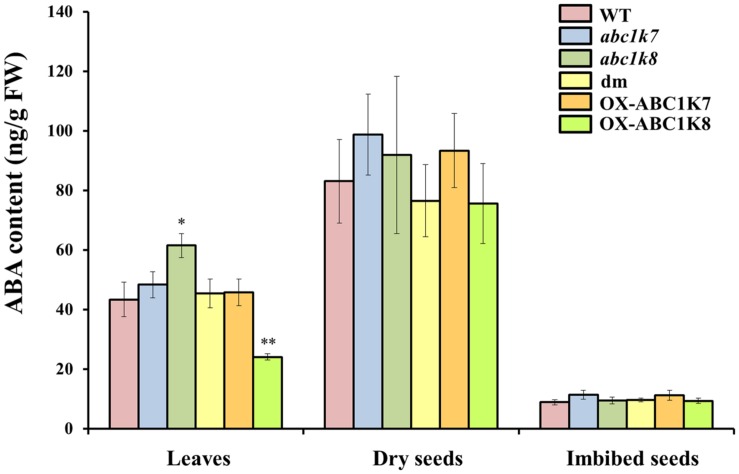
**Analysis of the ABA content in plant tissues.** The ABA content was quantified in leaves, dry seeds and imbibed seeds of WT, mutant and overexpressing plants grown under standard conditions. Each value represents the mean ± SD. Significant differences relative to the WT are shown as follows: ^∗^*P* < 0.05 and ^∗∗^*P* < 0.01.

The results described above suggested that *ABC1K7* and *ABC1K8* are likely to play a role in ABA signaling. Therefore, the endogenous ABA content was measured in the leaves, dry seeds, and imbibed seeds (24 h) of each genotype to determine whether the observed phenotypes could be explained by differences in the availability of ABA (**Figure 6**). We found no differences in ABA content in the dry and imbibed seeds of the mutant and WT plants. The leaves of the *abc1k8* mutant contained significantly more ABA than WT leaves (*P* < 0.05). Moreover, ABA levels were measured after Cd and mannitol treatments. Following Cd treatment, both single and double mutants showed an increased ABA content in comparison to WT, while mannitol did not influence ABA accumulation in leaves (**Supplementary Figure S2**).

## Discussion

In bacteria, archaea, and eukaryotic mitochondria, ABC1K proteins are essential for the biosynthesis of coenzyme Q, which functions as an electron carrier in the respiratory chain, and as a lipid-soluble antioxidant (Poon et al., 2000; Do et al., 2001; Mollet et al., 2008; Tauche et al., 2008). The *A. thaliana* chloroplast proteome contains members of the ABC1K family, which are involved in the regulation of prenylquinone metabolism (Lundquist et al., 2012a) and stress responses (Jasinski et al., 2008; Gao et al., 2010; Gao Q.S. et al., 2011; Wang et al., 2011). Indeed, the role of ABC1K proteins in abiotic stress tolerance is well documented and the transcription of ABC1K genes is often modulated by oxidative stress and salt (Jasinski et al., 2008; Gao et al., 2010, 2012; Wang et al., 2011; Yang et al., 2012a,b).

The involvement of ABC1K7 and ABC1K8 in oxidative stress responses, isoprenyl lipid biosynthesis and iron distribution within the chloroplast has recently been demonstrated (Manara et al., 2014, 2015). The *abc1k7* and *abc1k8* single mutants and the *abc1k7/abc1k8* double mutant produce more ROS and antioxidants than WT plants even under standard growth conditions, and are less tolerant to ROS and oxidative stress (Jasinski et al., 2008; Manara et al., 2014). These mutants also have a different polar lipid composition of chloroplast membranes in comparison to WT plants (Manara et al., 2015). Nevertheless, the precise biological role of these proteins is unclear because the *abc1k7* and *abc1k8* mutants show no changes in growth or development under standard conditions and the only visible phenotype is the pale green leaves of the *abc1k8* mutant line (Jasinski et al., 2008; Manara et al., 2014).

Even though ROS cause extensive cellular damage at high concentrations (Van Breusegem and Dat, 2006), they play an important physiological role in ABA signaling during seed development, maturation and dormancy, germination, seedling growth and seed aging (Liu et al., 2010). The transition from a quiescent to a vital seed is also associated with the generation of ROS (Gomes and Garcia, 2013). Antioxidant defense mechanisms usually maintain the intracellular concentration of ROS at a low level, known as the oxidative window, rather than eliminating them completely, so that their physiological functions can be executed (Bailly et al., 2008). In particular, the roles of ROS in seed dormancy, germination and the control of stomata movements depend on their interactions with phytohormones such as ABA (Neill et al., 2008; Simontacchi et al., 2013). Due to the fact that *abc1k7* and *abc1k8* showed an imbalanced cell redox state, we investigated the role of ABC1K7 and ABC1K8 in the regulation of ABA signaling to gain insight into their biological functions.

The *ABC1K7* and *ABC1K8* genes were both induced by ABA treatment and downregulated when endogenous ABA synthesis is inhibited by applying NDGA, suggesting that endogenous ABA content modulates their expression and that ABC1K7 and ABC1K8 proteins probably have a physiological role in ABA signaling. Considering the involvement of ABA in responses to abiotic stress, and taking into account that other ABC1K genes have been characterized by abiotic stress responsiveness (Wang et al., 2011, 2013; Lundquist et al., 2012a; Yang et al., 2012a), we investigated the effect of a variety of abiotic stresses, such as Cd treatment, cold and mannitol treatment, on the expression of *ABC1K7* and *ABC1K8*. We found that both genes are upregulated in response to the stress: both *ABC1K7* and *ABC1K8* show a substantial and clear upregulation upon Cd and mannitol exposure lasting also 24 h after the treatment, while cold stress imposes an early response. These results indicate that, similarly to other ABC1K proteins, also ABC1K7 and ABC1K8 have a role in response to abiotic stress also mediated by ABA signal transduction.

We investigated the basis of these interactions in more detail by measuring the ABA content of seeds in mutant plants and WT controls. There were no genotype-dependent differences in ABA content in the dry and imbibed seeds. This, together with the fact that both *ABC1K7* and *ABC1K8* mRNAs are modulated by ABA content, suggests that in these organs the *abc1k7*, *abc1k8*, and *abc1k7/abc1k8* mutations affect the perception of ABA. It appears that the loss of ABC1K7 and/or ABC1K8 activity increase ABA sensitivity. Indeed, even though the frequency of germination in the *abc1k7*, *abc1k8* and *abc1k7/abc1k8* mutants was similar to WT plants in the absence of ABA, it was delayed in mutant lines upon exposure to ABA. These differences were mostly eliminated following prolonged exposure to ABA, although the frequency of germination remained lower in the *abc1k7/abc1k8* double mutants at the highest ABA concentrations tested. Considering that ABA is a key mediator of abiotic stress responses (Himmelbach et al., 2003; Hubbard et al., 2010), we also tested the germination frequency of *abc1k7*, *abc1k8*, and *abc1k7/abc1k8* mutant lines under osmotic and salt stress. The presence of either mannitol or sodium chloride progressively inhibited germination in mutant lines. The ABA-sensitive phenotype of these mutants regarding seed germination and the upregulation of *ABC1K7* and *ABC1K8* upon abiotic stress, indicate that these ABC1K kinases may participate in ABA signaling during germination and play a role in the development of tolerance toward osmotic stress and salt stress.

Considering the expression of key players involved in the ABA signal transduction pathway, *HAB1* and *ABI1* genes were upregulated in the *abc1k7*, *abc1k8*, and *abc1k7/abc1k8* double mutant even in standard growth conditions, and overexpressed upon ABA treatment. Their gene products are Type 2C protein phosphatases (also known as PP2Cs) which constitutively block ABA signal transduction and expression of ABA-induced genes (Umezawa et al., 2010). Once abiotic stresses or developmental cues up-regulate endogenous ABA (or in case of exogenous ABA application), ABA receptors such as PYR/PYL/RCAR bind ABA and interact with PP2C, inhibiting its activity of negative regulator. This results in the activation of the ABA-induced gene expression (Umezawa et al., 2010). Plants overexpressing PP2C proteins are characterized by ABA insensitivity, while loss-of-function mutants show increased ABA sensitivity, in terms of germination rate upon mannitol, NaCl or ABA treatment (Saez et al., 2004). Considering that in *abc1k7*, *abc1k8* and *abc1k7/abc1k8, HAB1* and *ABI1* resulted up-regulated in both standard growth conditions and upon ABA treatment, the hypersensitivity to ABA, mannitol and NaCl of seed germination in *abc1k7*, *abc1k8*, and *abc1k7/abc1k8* points to a perturbed ABA sensing in these lines.

Under standard conditions, stomatal opening was inhibited in the *abc1k7*, *abc1k8* and *abc1k7/abc1k8* mutants compared to WT plants, while no stomatal movements were observed in the *abc1k7*, *abc1k8*, and *abc1k7/abc1k8* mutants upon ABA treatment. This may be explained taking into consideration that *abc1k7*, *abc1k8* and *abc1k7/abc1k8* mutants showed an imbalanced leaf redox state (Jasinski et al., 2008; Manara et al., 2014), which may influence ABA-mediated stomatal movements, e.g., H_2_O_2_ mediates the ABA-driven induction of Ca^2+^-permeable, non-selective cation current channels (Pei et al., 2000; Murata et al., 2001; Kwak et al., 2003). This, again, points to a higher sensitivity of *abc1k7* and *abc1k8* plants to the endogenous ABA.

Interestingly, the low germination rate and increased stomatal closure following treatment with ABA observed in *abc1k7*, *abc1k8*, and *abc1k7/abc1k8* double mutants may also reflect the greater abundance of OPDA-esterified galactolipids observed in these mutant lines (Manara et al., 2015). Indeed, galactolipids conjugated with OPDA and dnOPDA may allow the storage of reactive oxylipins (Böttcher and Weiler, 2007) and free OPDA may acts synergistically with ABA to inhibit germination and regulate stomatal closure (Dave et al., 2011; Savchenko et al., 2014).

Abscisic acid content was investigated also in term of expression analysis of key members of ABA metabolism. This shows an upregulation, in mutant plants, of *NCED3* and *DTX50*, involved in ABA synthesis and transport respectively, which may locally affect ABA content. ABA content was measured in leaves of mutant plants and WT: a moderate increase in ABA abundance was detected only in leaves of *abc1k8* in control conditions. It may be that ABA distribution inside the leaf, considering stomata guard cell as a target site, may be affected and accounting for the detected stomata closure. Alternatively, the increased ROS and SOD activity characteristic of the mutant lines (Manara et al., 2014) may enhance H_2_O_2_ production, which can in turn contribute to stomatal closure without ABA application (Zhang et al., 2001).

The role of ABC1K7 and ABC1K8 in ABA signaling is also supported by the comparative transcriptional analysis of six different ABA-induced genes in mutant and WT plants. Other than the already mentioned *ABI1* and *HAB1*, two LEA genes (*Cor15b* and *ERD10*) were upregulated in mutant backgrounds after ABA application. *Cor15b* is induced by low temperatures and exogenous ABA (Wilhelm and Thomashow, 1993) and *ERD10* is induced by temperatures, exogenous ABA and dehydration (Kiyosue et al., 1994). The two stress-inducible KIN genes (*KIN1* and *KIN2*) were induced more strongly in the *abc1k8* and *abc1k7/abc1k8* mutant plants than in WT, following 5 h of ABA treatment, whereas the *abc1k7* mutant behaved in a similar manner to WT. These genes are known to be induced by low temperatures and exogenous ABA (Kurkela and Borg-Franck, 1992; Wang et al., 1995). The induction of all six genes was short-lived and differences between mutants and WT plants had diminished after 24 h exposure, although in the case of *KIN1* and *KIN2* the transcript levels fell below WT levels after 24 h.

In addition to abiotic stress, ABA is also a positive regulator of leaf senescence (Lee et al., 2011) and exogenous ABA application induced senescence, associated with the loss of chlorophyll (Jaradat et al., 2013). When floated on ABA solution, detached leaves from *abc1k7*, *abc1k8*, and *abc1k7/abc1k8* mutant showed typical symptoms of yellowing and chlorosis, while WT leaves showed a limited senescence response. These distinct phenotypes were confirmed by measuring the chlorophyll content, which also highlighted lower chlorophyll content in standard growth conditions (as reported previously, Manara et al., 2014). Among the mutant plants, upon ABA treatment *abc1k7* leaves showed the most severe symptoms of ABA-induced senescence and the strongest expression of the senescence-associated *SAG12* (Grbić, 2003). These results are supported by the recent observations that *ABC1K7* is induced by and potentially involved in senescence, which correlates with higher levels of oxidative stress and ROS production (Lundquist et al., 2012b) and that *abc1k7* is characterized by increased plastoglobule dimension (Manara et al., 2014). The plastoglobule proteome contains ABC1K7 and SAG in a senescence-associated protein cluster, which reveals senescence-associated and senescence-induced proteases, and two chlorophyll degradation enzymes (Lundquist et al., 2012b). The latter are coexpressed with ABC1K7 and SAG, explaining the loss of chlorophyll content and the stronger induction of *SAG12* in the *abc1k7* mutant in response to ABA. Therefore, our data suggest that *ABC1K7* and *ABC1K8* may be involved in alleviating leaf senescence, which is enhanced in mutant plants. Moreover, the accumulation of OPDA-containing galactolipids, observed in *abc1k7*, *abc1k8*, and *abc1k7/abc1k8* mutant plants (Manara et al., 2015), may contribute to the senescence-like phenotype, the reduction of chlorophyll content and the induction of *SAG12* in response to ABA treatment.

## Conclusion

The presented findings point to a role of ABC1K7 and ABC1K8 in handling the metabolism activated in response to abiotic stress (e.g., drought) or particular physiological process (i.e., senescence), which involve a cross-talk between ABA and ROS signaling. It could be reasonable to speculate that these proteins may play a role in the ABA signal transduction pathway or ROS production, being located in the plastid, the principal cellular compartment deputed to these anabolic reactions. However, further research is in progress to shed light on the mechanism of action of ABC1K7 and ABC1K8 together with other ABC1K proteins in *Arabidopsis*.

## Author Contributions

AM, GDC, and AF designed the research, discussed the results and wrote the article; AM and GDC performed the research experiments. All the authors listed approved the work for publication.

## Conflict of Interest Statement

The authors declare that the research was conducted in the absence of any commercial or financial relationships that could be construed as a potential conflict of interest.

The reviewer MX and handling Editor declared their shared affiliation, and the handling Editor states that the process nevertheless met the standards of a fair and objective review.

## Abbreviations

ABA: abscisic acid
ABC1K: activity of *bc1* complex kinase
AtACDO1: ABC1-like kinase related to chlorophyll degradation and oxidative stress
AtOSA1: oxidative stress related ABC1-like protein
AtSIA1: salt induced ABC1 kinase 1
LEA: late embryogenesis abundant
ROS: reactive oxygen species
WT: wild type
