# Supplementary material for: The Role of the Atypical Kinases ABC1K7 and ABC1K8 in Abscisic Acid Responses
Source: Front Plant Sci. 2016 Mar 24;7:366. doi: 10.3389/fpls.2016.00366 (PMC4805650; doi:10.3389/fpls.2016.00366)
Supplement: Supplementary file 1 [file Image_1.PDF]

Supplementary Material

The role of the atypical kinases ABC1K7 and ABC1K8 in abscisic acid responses

Giovanni DalCorso, Anna Manara, and Antonella Furini*

*** Correspondence:** Antonella Furini: antonella.furini@univr.it

1 Supplementary Materials and Methods

Analysis of expression of gene involved in ABA metabolism

Leaf samples were collected from three-week old wild type, *abc1k7*, *abc1k8* and *abc1k7/abc1k8* plants kept in standard growth conditions and from the same lines subjected to de-hydration under a laminar flow hood for 6 hours. Total RNA was extracted from fresh tissue using TRIzol reagent (Invitrogen, Karlsruhe, Germany). After DNase treatment, first-strand cDNA was synthesized using SuperScript™ III Reverse Transcriptase (Invitrogen). Real-time RT-PCR (40 amplification cycles) was carried out using the StepOnePlus Real-Time PCR System (Applied Biosystems, Foster City, CA, USA) with Platinum® SYBR® Green qPCRSuperMix UDG (Invitrogen). Each reaction was carried out in triplicate and melting curves were analyzed to ensure the amplification of a single product. Quantitative data were normalized to the mean of two endogenous reference genes: actin2/8 (*At3g18780/At1g49240*; primers 1 and 2) and ubiquitin 5 (*At3g62250*; primers 3 and 4). The $2^{-\Delta\Delta CT}$ method for the analysis of relative gene expression levels was used to organize the data (Livak and Schmittgen, 2001). Primers used were specifically designed on *At1g69850* (AIT1F-RTGAAGTGGAAGAAGAGGTCTC, AIT1R-RT GCATTCGCCAAATACGCTAG), *At3g14440* (Nced3F-RT GCTGCGGTTTCTGGGAGAT, Ncde3R-RTAGGAGTGTGAAGCGCAGAT), *At2g27150* (AAO3F-RT GCTTCCTGGCATTTGGTTCT, AAO3R-RTTTGCCAG GGACTATGATTCC), *At5g52050* (DTX50F-RT GCAAACATCACCGGATACTC, DTX50R-RT ACGCGAGCTTCTT GTCCTGT) and their efficiency was determined using LinRegPCR v2012.2 (Ramakers et al. 2003). (see **Supplementary Figure 1**).

2 Supplementary Figures and Tables

2.1 Supplementary Figures

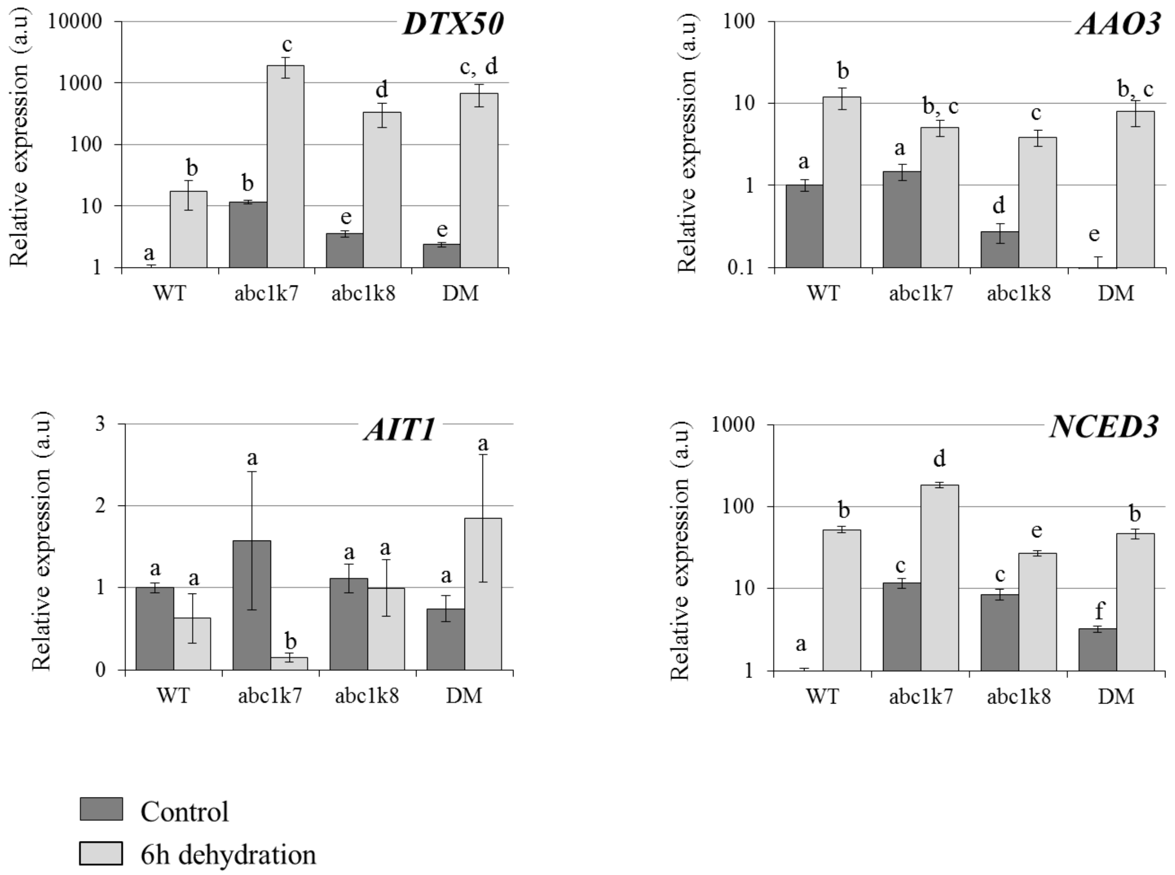

Supplementary Figure 1. Analysis of RNA abundance of genes involved in ABA metabolism by Real Time PCR. The analysis was performed in dehydrated plants and results were compared to non-treated control plants, as described in Supplementary Material and Methods. In the histograms, values sharing a common letter are not significantly different at P < 0.05.

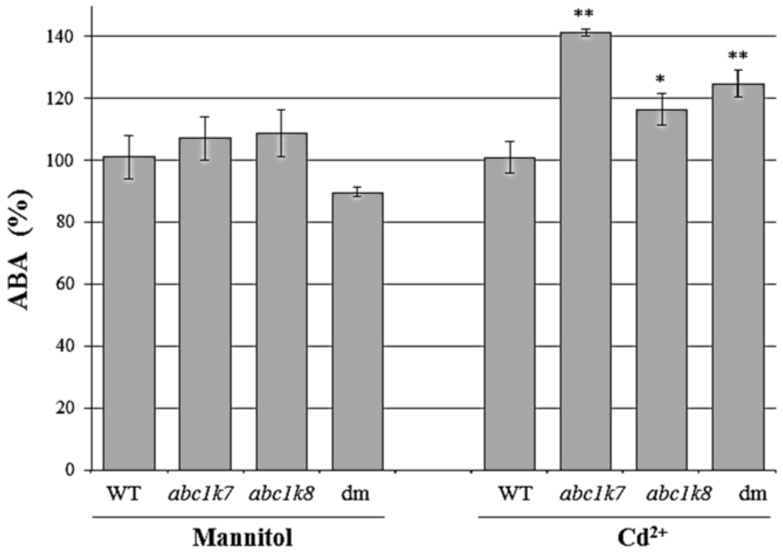

Supplementary Figure 2. The ABA content was quantified in leaves of WT and mutant plants upon 7-days treatments with mannitol and cadmium. Each value represents the mean ± SD. Significant differences relative to the wild type are shown as follows: * (P<0.05) and ** (P<0.01).
